# Case Report: Viral Shedding for 60 Days in a Woman with COVID-19

**DOI:** 10.4269/ajtmh.20-0275

**Published:** 2020-04-27

**Authors:** Junyao Li, Lin Zhang, Baihui Liu, Debiao Song

**Affiliations:** 1 Department of Respiratory and Critical Care Medicine, The Second Hospital of Jilin University, Changchun, People’s Republic of China;; 2 Department of Emergency and Critical Care Medicine, The Second Hospital of Jilin University, Changchun, People’s Republic of China

## Abstract

Novel coronavirus disease (COVID-19) caused by severe acute respiratory syndrome-coronavirus-2 (SARS-CoV-2) has become a public health emergency of international concern. This was first noted in Wuhan, Hubei Province, China, and since then has become widespread globally. We report a 71-year-old woman with documented viral shedding (based on reverse transcription–polymerase chain reaction (RT-PCR) testing) of SARS-CoV-2 for 60 days from the onset of symptoms (55 days from her first positive test and 36 days after complete resolution of symptoms). This is to our knowledge the longest duration of viral shedding reported to date. This case demonstrates that viral shedding after COVID-19 diagnosis can be prolonged.

## INTRODUCTION

In December 2019, an outbreak of severe acute respiratory syndrome-coronavirus-2 (SARS-CoV-2) infection was detected in Wuhan, China. The extensive spread of SARS-CoV-2 has led to a massive pandemic, associated with substantial morbidity and mortality.^1^ Novel coronavirus disease (COVID-19) has spread to more than 200 countries, and the death toll remains very high.^2^ According to some retrospective analyses, the median duration of viral shedding was 12–20 days from illness onset,^3–5^ whereas the longest duration recorded has been 49 days.^6^ We present a case of COVID-19 with viral shedding for 60 days from the onset of symptoms.

## CASE PRESENTATION

**Figure 1.**
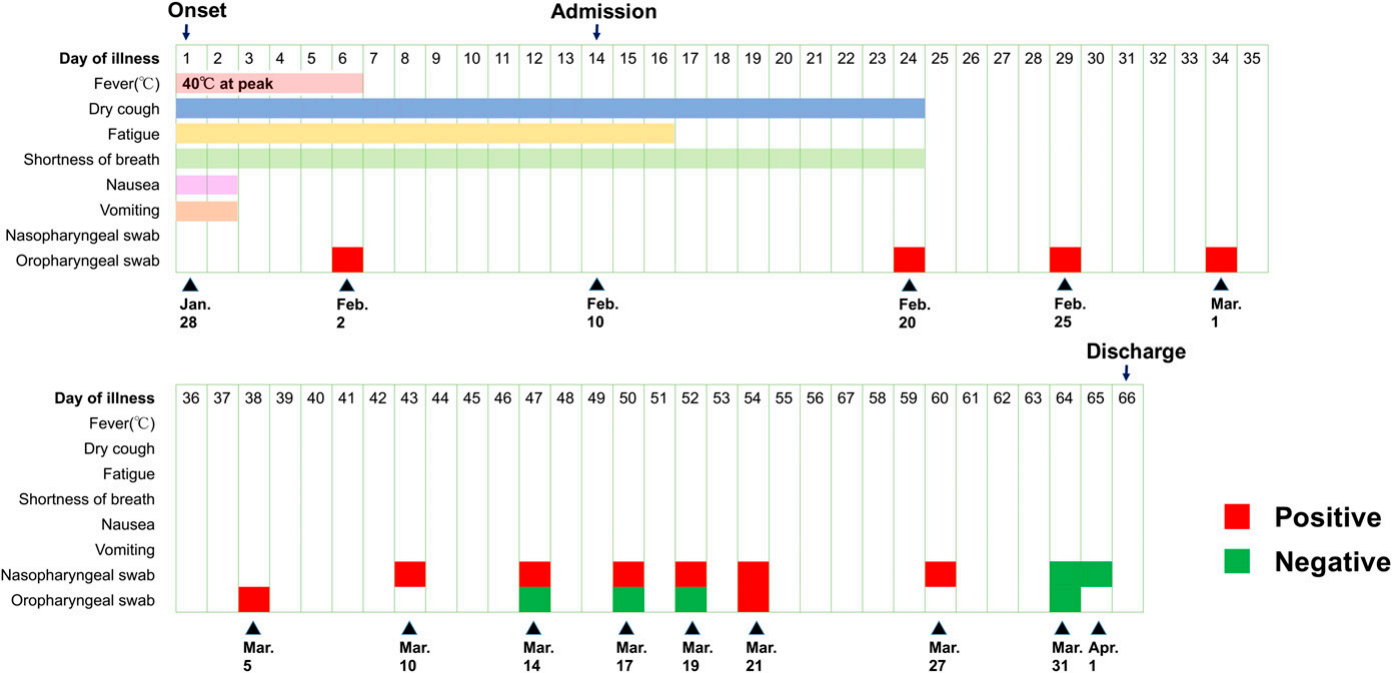
Dynamic assessment of symptoms and severe acute respiratory syndrome-coronavirus-2 reverse transcriptase–PCR test results over time.

On February 10, 2020, a 71-year-old woman who lives in Wuhan was admitted to hospital with a 14-day history of illness, 9 days after a positive reverse transcriptase (RT)-PCR test for SARS-CoV-2 (Figure 1). The patient reported a history of intermittent fever (highest 40.0°C) for the initial 6 days of illness, and dry cough, fatigue, and shortness of breath, which were worse at night for 14 days. She also had nausea, vomiting, and diarrhea for the first 2 days of illness. Because of increasing dyspnea, a computed tomography (CT) scan was conducted on January 30, 2020 (illness day 3); this showed multiple small patchy shadows and interstitial changes, consistent with viral pneumonia. A subsequent oropharyngeal swab RT-PCR test was positive for COVID-19 on February 2, 2020 (illness day 6). The patient was treated as an outpatient with moxifloxacin hydrochloride and Lianhuaqingwen (a Chinese herbal medicine) from days 1 to 13 of illness.

Apart from a history of penicillin allergy, the patient was healthy and a nonsmoker. Despite resolution of fever after day 6 of illness, her symptoms of dry cough and fatigue persisted, and shortness of breath worsened, leading to hospital admission on day 14 of illness. On physical examination, a temperature of 36.6°C, blood pressure of 139/94 mmHg, pulse of 84 per minute, respiratory rate of 20 per minute, and oxygen saturation of 94% breathing room air were recorded. Lung auscultation revealed slightly wet rales from both the lungs, but no dry rales were noted. No obvious abnormalities were found on the rest of the examination.

**Figure 2.**
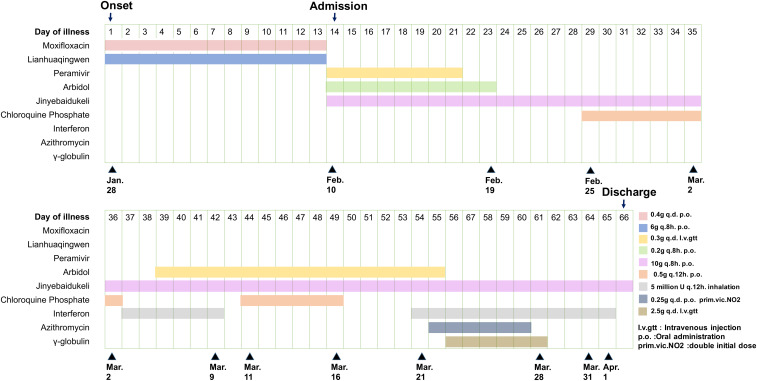
Medications administered.

**Figure 3.**
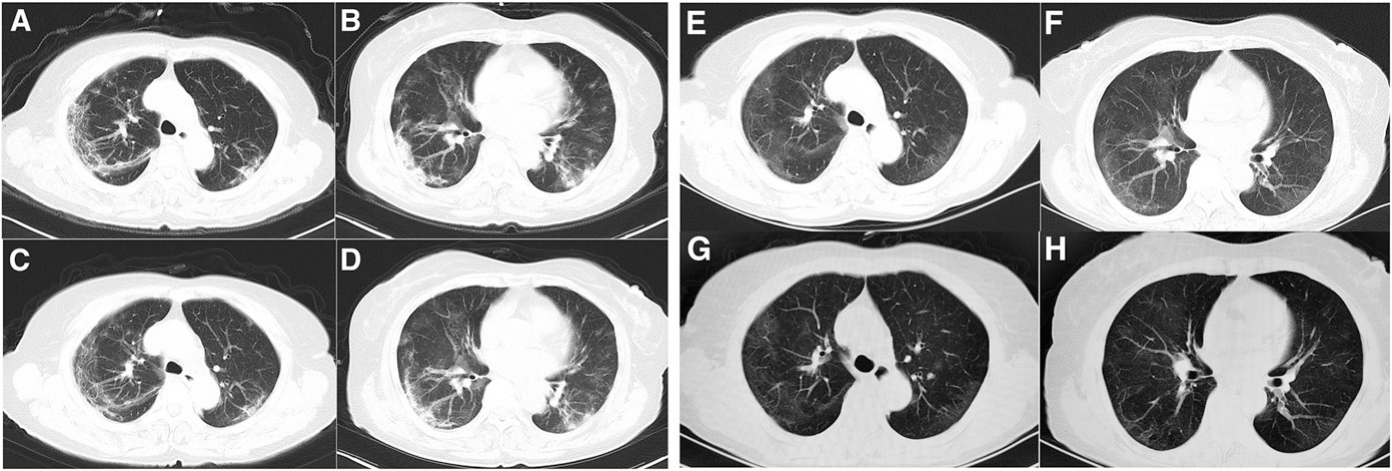
Chest computed tomography images. The images show multiple patchy shadows and bilateral subpleural ground-glass opacities and infiltrates, with improvement over time. (**A** and **B**): February 11; (**C** and **D**): February 17; (**E** and **F**): March 6; (**G** and **H**): March 21 (illness day 15, 21, 39, and 54, respectively).

After admission, the patient received antiviral treatment including peramivir–sodium chloride injection and arbidol tablets according to Chinese Clinical Guidance for COVID-19 Pneumonia Diagnosis and Treatment.^7^ The patient was kept at bed rest with continuous low flow oxygen, with treatment as shown in Figure 2. Laboratory results are shown in Table 1. Computed tomography scan on hospital day 2 (illness day 15) showed multiple patchy shadows, ground-glass opacities, and infiltrates in subpleural areas bilaterally (Figure 3A and B).

**Table 1:** Clinical laboratory result

Measure	Reference range	Illness day 14	Illness day 31	Illness day 45	Illness day 61
White cell count (×10^9^/L)	3.50–9.50	4.47	3.39	3.34	4.2
Absolute neutrophil count (×10^9^/L)	1.80–6.30	3.18	1.91	1.70	3.11
Absolute lymphocyte count (×10^9^/L)	1.10–3.20	0.66	0.79	1.02	0.72
Absolute monocyte count (×10^9^/L)	0.10–0.60	0.53	0.44	0.45	0.35
Red cell (×10^9^/L)	3.80–5.10	3.72	3.71	3.60	3.93
Hematocrit (%)	35.0–45.0	34.3	34.9	33.2	35.4
Hemoglobin (×10^9^/L)	115.0–150.0	118	117	112	123
Platelet count (×10^9^/L)	125.0–350.0	433	218	229	159
Alanine aminotransferase (U/L)	≤ 33	18	42	14	–
Aspartate aminotransferase (U/L)	≤ 32	19	43	17	–
Total protein (g/L)	64–83	69.6	67.4	63.7	–
Albumin (g/L)	35–53	32.5	37.1	36.4	–
Total bilirubin (µmol/L)	≤ 21	4.3	7.7	5.2	–
Alkaline phosphatase (U/L)	35–105	57	58	53	–
Blood urea nitrogen (mmol/L)	3.1–8.8	3.40	4.70	4.00	–
Creatinine (µmol/L)	45–84	50	64	59	–
Lactate dehydrogenase (U/L)	135–214	284	180	220	15
Potassium (mmol/L)	3.50–5.10	3.85	4.26	4.04	–
Sodium (mmol/L)	136–145	142.7	140.6	140.9	–
Chloride (mmol/L)	99–110	103.0	101.3	103.7	–
Calcium (mmol/L)	2.15–2.57	2.04	2.50	2.39	–
Glucose (mmol/L)	4.11–6.05	5.83	4.84	5.00	–
Procalcitonin (ng/mL)	< 0.05	0.04	0.08	0.02	–
Erythrocyte sedimentation rate (mm/H)	< 20.00	88	23	16	54
C-reactive protein (mg/L)	< 1	7.6	1.7	1.6	–
Prothrombin time (seconds)	11.5–14.5	12.6	12.6	13.8	–
Fibrinogen (g/L)	2.00–4.00	5.88	3.47	3.22	–
D-dimer (µg/mL)	< 0.5	1.99	2.08	1.53	–
Fer protein (µg/L)	15–150	–	228.4	165.2	–
Total T lymphocyte (/µL)	955–2860	–	–	–	449
T-lymphocyte CD3^+^CD4+ (/µL)	550–1440	–	–	–	214
T-lymphocyte CD3^+^CD8^+^ (/µL)	320–1250	–	–	–	180

From day 4 (illness day 17) of hospitalization, the patient had no fatigue but had occasional dry cough and shortness of breath after activities. On day 5 through 11 of hospitalization (illness days 18–24), her persisting symptoms improved gradually. From hospital day 12 (illness day 25), the patient was asymptomatic, with an oxygen saturation of 97% on room air, but she remained hospitalized based on local policies. Repeat CT scans showed gradual improvement in lung abnormalities (Figure 3). Repeat SARS-CoV-2 tests of nasopharyngeal and/or oropharyngeal swabs were positive through day 60 of illness, with negative tests on days 64 and 65 (Figure 1). Sinus CT scan on hospital day 41 showed no obvious abnormalities. Given her prolonged viral shedding, the patient received γ-globulin treatment from illness days 56 to 61. The patient was discharged from the hospital on April 2, 2020, 66 days from the onset of illness, after consecutive nasopharyngeal swabs and an oropharyngeal swab tested negative for SARS-CoV-2.^8^

## DISCUSSION

We report persistent shedding of SARS-CoV-2, based on RT-PCR testing, for 60 days from the onset of typical symptoms of COVID-19 in a 71-year-old Chinese woman. Researchers have reported that both symptomatic and asymptomatic patients can have similar viral loads, suggesting a transmission potential for asymptomatic or minimally symptomatic patients.^9,10^ Our case recovered from a fairly mild episode of COVID-19, with no reported symptoms after illness day 24, but viral shedding persisted for another 36 days. This is to our knowledge the longest duration of SARS-COVID-2 shedding reported to date.

There have been several reports on infections in close contacts of COVID-19 patients even after apparent clinical recovery of the source patients.^11^ Transmission has also been documented from asymptomatic or minimally symptomatic patients.^12,13^ Similar to previously reported cases, we report prolonged viral shedding after recovery from COVID-19. It remains unclear whether prolonged shedding is associated with prolonged infectivity. Investigation of the importance of the “shedding window” after clinical recovery from COVID-19 is of high priority.

In summary, we reported a case of COVID-19 with viral shedding for 60 days from illness onset and, more importantly, persistent viral shedding 36 days after resolution of symptoms, suggesting that asymptomatic, mildly symptomatic, and recently recovered patients may require prolonged isolation. In addition, further studies based on larger cohorts would help to characterize the duration of viral shedding and infectivity.
